# Exploring the effectiveness of methyl salicylate and attractive traps in dispersing and recapturing glasshouse whiteflies: advances in push–pull strategy

**DOI:** 10.1002/ps.70021

**Published:** 2025-06-27

**Authors:** Maria Athanasiadou, Rainer Meyhöfer

**Affiliations:** ^1^ Applied Entomology Institute of Horticultural Production Systems, WG Phytomedicine, Leibniz Universität Hannover Hannover Germany

**Keywords:** integrated pest management (IPM), visual manipulation, chemical ecology, herbivore‐induced plant volatiles (HIPVs), insect repellency, light‐emitting diodes (LEDs)

## Abstract

**BACKGROUND:**

Methyl salicylate (MeSa), a plant defense elicitor and whitefly deterrent, has been shown to reduce the settlement of the glasshouse whitefly (*Trialeurodes vaporariorum*) on host plants. This study aimed to evaluate the dispersal effect of MeSa on whiteflies settled on tomato plants and assess the efficacy of yellow sticky traps (YST) and green light‐emitting diode (LED)‐enhanced YST (green LED trap (GLT)) in recapturing dispersed whiteflies. These findings form the basis for developing a push–pull strategy to enhance whitefly control.

**RESULTS:**

Initial experiments tested varying MeSa concentrations, revealing that higher concentrations significantly increased whitefly dispersal, with the highest concentration causing nearly four times more dispersal compared to the control. Subsequent scaling‐up experiments evaluated the dispersal effect of the most effective MeSa concentration from the first experiment, in presence of a YST or a GLT. Results revealed that MeSa significantly increased whitefly dispersal from plants within just 2 h, with nearly 50% dispersal after 24 h and 95% recapture rate on the traps. The GLT consistently captured more whiteflies than the YST, and both traps showed enhanced catching efficacy in the presence of MeSa compared to the control.

**CONCLUSION:**

These findings highlight the potential of combining MeSa as whitefly dispersal factor (push) with green LED‐enhanced traps as attractive factor (pull) in push–pull strategies to improve targeted whitefly control in glasshouse crops, while reducing reliance on pesticides. © 2025 The Author(s). *Pest Management Science* published by John Wiley & Sons Ltd on behalf of Society of Chemical Industry.

## Graphical abstract

Methyl salicylate causes dispersal of whiteflies from tomato plants (push), and enhances the catching efficacy of an attractive green‐light‐emitting diode (LED)‐enhanced yellow sticky trap (pull), showing potential for a novel push–pull system.

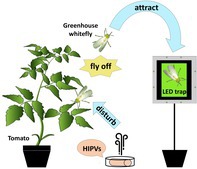

## 1 INTRODUCTION

Push–pull strategies employ behavioral manipulation of insects by combining repelling stimuli that deter pests from a crop (push) with attractive stimuli that draw them towards a specific site (pull), for example a trap, thereby effectively controlling their population within the crop. The efficacy of push–pull strategies is improved by simultaneously deploying these behavioral stimuli. Such strategies can significantly reduce pest population density and minimize the need for insecticides in various agricultural systems.^1^ They include use of visual and olfactory stimuli to repel and attract pests. An example of a natural push–pull system involves intercropping *Desmodium* plants with maize to repel stem borers and fall armyworms through volatile emissions, while Napier grass, planted around the maize field, attracts those pests more effectively than the maize itself.^2^, ^3^, ^4^ In our research, we sought to develop a push–pull method using a technical approach by targeting glasshouse whiteflies, *Trialeurodes vaporariorum* (Westwood), one of the most prominent pests of glasshouse crops in Europe.^5^, ^6^ More specifically, we used synthetic olfactory cues to deter glasshouse whiteflies from their host plants, in order to lure them towards a light trap. After landing on the trap, whiteflies can get eliminated by use of technological means, such as identification by a camera system and application of low power, lethal, laser beams, a technique developed in the joint research project ‘LichtFalle’, funded by the German Ministry of Food and Agriculture.^7^ Taking into account that a cue can be classified as repellent when it triggers an organism to move away from its host,^8^ and that disruption of feeding activity by deterrent stimuli can be a type of insect repellence,^9^ we considered repellent a stimulus that pushes an insect away from its host before as well as after landing onto it.

Apart from visual and gustatory cues, whiteflies rely largely on olfactory cues for locating and assessing suitable host plants,^10^ as they possess a full equipment of olfactory receptors.^11^ Once a whitefly detects a potential host plant using visual cues, olfactory signals further guide its foraging behavior. These olfactory cues are primarily volatile organic compounds (VOCs) emitted by plants,^12^ and they play an important role in whiteflies ecological interactions, such as feeding and oviposition,^10^ while providing them with critical information about the host's condition.^13^ Plants emit specific VOCs in response to health or stress levels, which can signal the activation of defense mechanisms.^14^ For example, when under attack, plants release volatiles that attract whiteflies’ natural enemies, such as parasitic wasps or predatory insects.^15^ These same volatiles can deter whiteflies by indicating that a plant has initiated its defensive responses and by signaling poor plant quality.^16^ Ecologically, whiteflies can be effectively deterred by several VOCs. Limonene, a citrus‐derived compound, showed reduced whitefly populations on treated plants,^14^ while caryophyllene and hexanoic acid, released by plants under herbivore attack, decreased whitefly colonization.^17^, ^18^, ^19^, ^20^ Linalool and (*E*)‐*β*‐farnesene, terpenes emitted by plants in response to stress, indicate active plant defenses or mimic alarm signals, respectively. Consequently, they repel whiteflies by disrupting their olfactory‐guided behaviors and suggesting a hostile environment.^18^, ^19^ Furthermore, signaling compounds like methyl jasmonate (MeJa) and methyl salicylate (MeSa) are integral to plant defense pathways. MeJa triggers plant defenses that can deter herbivores by signaling the activation of anti‐feeding and anti‐oviposition responses.^21^ MeSa is a methyl ester of salicylic acid (SA), a naturally occurring organic compound that serves as a plant volatile and plays a critical role in plant defense mechanisms.^22^ Aphid and whitefly feeding induces SA‐related defenses, prompting tomato plants to release herbivore‐induced plant volatiles (HIPVs), such as MeSA, which triggers systemic acquired resistance (SAR).^21^, ^23^, ^24^, ^25^, ^26^ This process activates defense mechanisms across the plant, leading to the production of proteins and compounds that enhance the plant's resistance to further attacks and render it less attractive for feeding and reproduction.^27^, ^28^, ^29^, ^30^, ^31^ MeSA not only repels whiteflies, but also attracts beneficial insects such as predators and parasitoids that prey on herbivores, thus playing a dual role in pest management.^15^ It has been suggested that MeSA may also function as a long‐distance signaling molecule within plants,^32^ as demonstrated in tobacco.^33^ Previous studies indicated that tomato plants treated with MeSa were repellent to whiteflies, as exposure to MeSa enhanced the expression of defensive genes by the plant, and were attractive to the parasitoid *Encarsia formosa*.^16^, ^34^ Furthermore, MeSA applications in tomato crops reduced whitefly populations and increased yield, likely due to the activation of plant defenses.^35^ Additionally, MeSA‐based lures in strawberry and cucumber plants attracted beneficial insects like Chrysopidae, *Orius tristicolor* and natural enemies of cotton aphids.^36^, ^37^ These studies were focused on the settling behavior of whiteflies on treated plants and, thus, can be implemented as a preventive strategy to reduce their settlement on crops. However, no studies could be found on the effect of MeSa on the behavior of whiteflies after they have colonized a host plant. Therefore, our study represents the first to examine the impact of this volatile on the behavior of the glasshouse whitefly settled on a host (push) and the effectiveness of a trap on the whitefly recapture (pull). Employing MeSa as a deterrent cue could potentially disturb whiteflies from their host plants, inducing take‐off behavior and increasing the likelihood of them landing on visually attractive traps. Studies on whitefly visual sensitivity demonstrated that this species display a ‘settling’ response on yellow‐reflecting surfaces, which led to the widespread use of yellow sticky traps (YSTs) to monitor populations and enable timely targeted interventions.^38^, ^39^, ^40^, ^41^, ^42^, ^43^ Further research revealed that whiteflies significantly preferred YSTs enhanced with green light‐emitting diodes (LEDs) compared to unlit YSTs, and they were more attracted to green than yellow light.^44^, ^45^, ^46^, ^47^ Therefore, in our study, we evaluated different concentrations of MeSa as a repelling cue on whiteflies settled on tomato leaves, and in a subsequent scaling‐up experiment, we tested the most effective MeSa concentration from the first experiment on whiteflies settled on tomato plants, in presence of a YST or a green LED‐enhanced YST (referred to as ‘green LED trap (GLT)’), in controlled conditions. Based on the literature, we hypothesized that MeSa would efficiently disturb whiteflies from their host, enhancing the capture rates of both traps, with the GLT demonstrating higher attractiveness compared to the traditional YST.

## 2 MATERIAL AND METHODS

### 2.1 Rearing of the glasshouse whiteflies

All glasshouse whiteflies (*T. vaporariorum*) used in this study were reared on tomato plants (*Solanum lycopersicum* L. cv. Brioso) in a gauze cage (90 cm × 60 cm × 60 cm) in a climate chamber at 23 ± 3°C, 50% ± 5% relative humidity (RH) and light/dark 16 h:8 h, at the Institute of Horticultural Production Systems, Leibniz Universität Hannover, Germany. For each experimental trial, about five adult whiteflies, without further sex identification, were carefully collected with an aspirator from the underside of leaves into an Eppendorf tube (1.5 mL) or a glass vial (height × diameter (*h* × *d*) = 5 cm × 2.5 cm), which was then placed into the arena of the first and second experiment respectively. All tomato plants were grown in pots (*h* × *d* = 10 cm × 12 cm) under glasshouse conditions before introduction to the rearing or the experiments.

### 2.2 Types of traps

**Figure 1:**
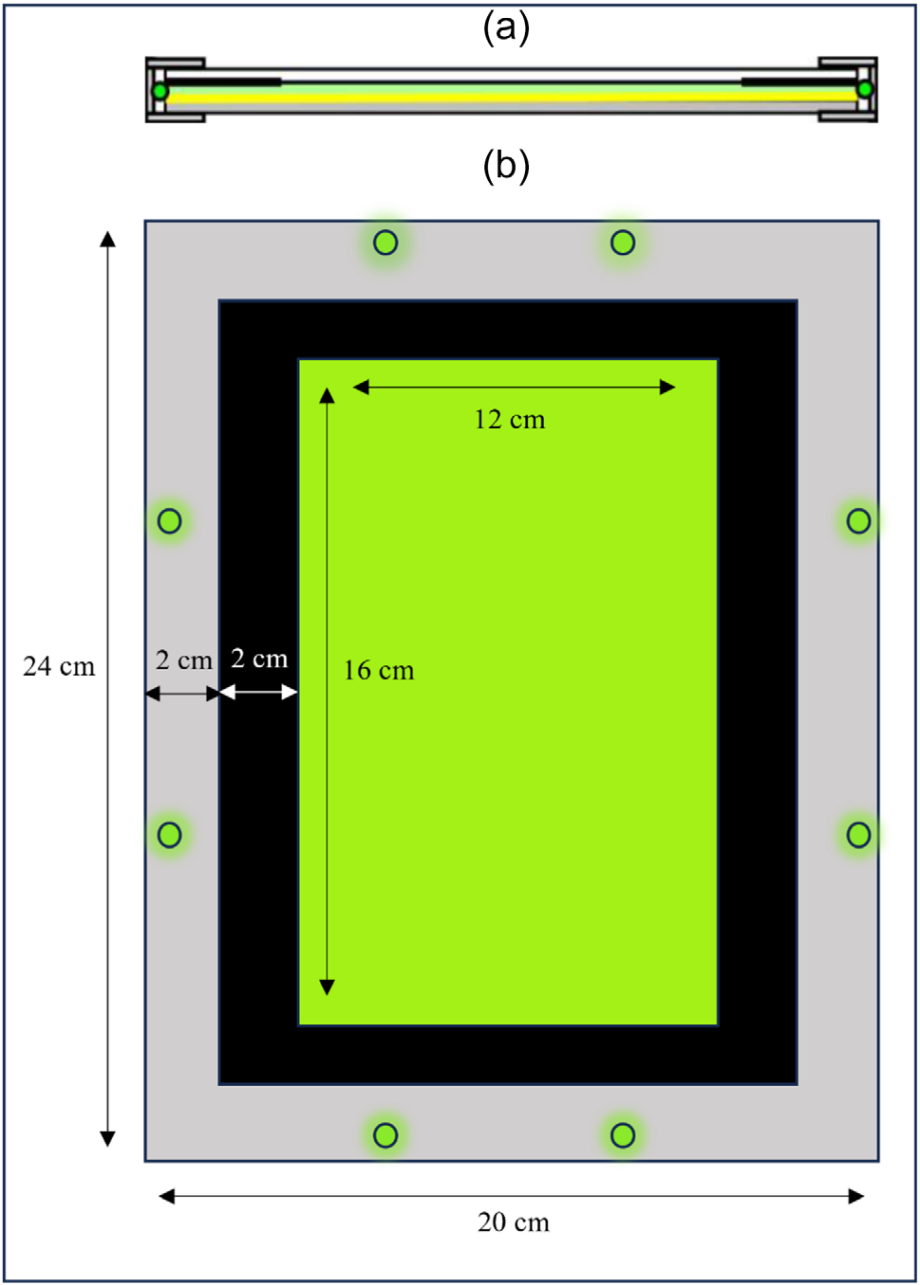
Scheme of the green light‐emitting diode (LED)‐enhanced yellow sticky trap. (a) Cross‐section showing the aluminum frame enclosing the green LEDs. All the layers from the bottom: polyvinyl chloride (PVC) plate, yellow card, LED scattering acrylic glass, black cardboard frame and transparent acrylic glass cover. (b) Top view scheme showing trapping surface, black cardboard frame and layout of green LEDs inside the aluminum frame. (based on Stukenberg 2018).^48^

Two types of traps were used in our experiment: a conventional YST and a GLT. The GLT was a trap designed by Stukenberg (2018)^48^ and already used by Athanasiadou *et al*.^47^: enclosed in an aluminum frame (24 cm × 20 cm) was a non‐sticky yellow card (22 cm × 18 cm) (IVOG Biotechnical Systems GmbH, Vogelsang, Germany) and eight green LED chips (520 nm, 1.0 cm × 1.0 cm, 1 W, 3 V, Nichia NCSG219BT‐V1; Luminotrix® LED‐Technik GmbH, Hechingen, Germany), facing inwards (two per side) (Fig. 1). The size of the illuminating area of the trap was 16 cm × 12 cm. Transparent plastic film (16 cm × 12 cm × 0.02 cm; Rico Design GmbH & Co. KG, Brakel, Germany) was attached on the top of the card and coated with insect glue (Insektenleim; Temmen GmbH, Hattersheim, Germany) for whitefly capturing. The intensity of the light trap was 5 μmol/m^2^/s and was measured with the LI 250 Light Meter and the LI 190 Quantum Sensor (LI‐COR Biosciences GmbH, Bad Homburg, Germany) 25 cm from the center of the trap (plant's position), in darkness. All green LEDs were used at maximum intensity, at 350 mA operating current. Regarding the conventional monitoring trap, a YST (IVOG Biotechnical Systems GmbH) was cut into the same size as the illuminating area of the GLT, and only one side of it was accessed by the whiteflies.

### 2.3 Experimental design and procedure

Two experiments were conducted in sequence to study the deterring effect of MeSa (Sigma Aldrich, Steinheim, Germany) on *T. vaporariorum* settled on a host, and the contribution of a trap on their recapture: a leaf experiment without a trap, and a scaling‐up plant experiment with a trap, both in a climate chamber, at 24 ± 2 °C, 60 ± 5% RH, light/dark 16 h:8 h, at the Institute of Horticultural Production Systems, Leibniz Universität Hannover, Germany. Both experiments were conducted on a bench covered with black plastic mulching film, 1.2 m below the fluorescent tubes of the chamber. In the following paragraphs, the specifics of each experiment are described.

**Figure 2:**
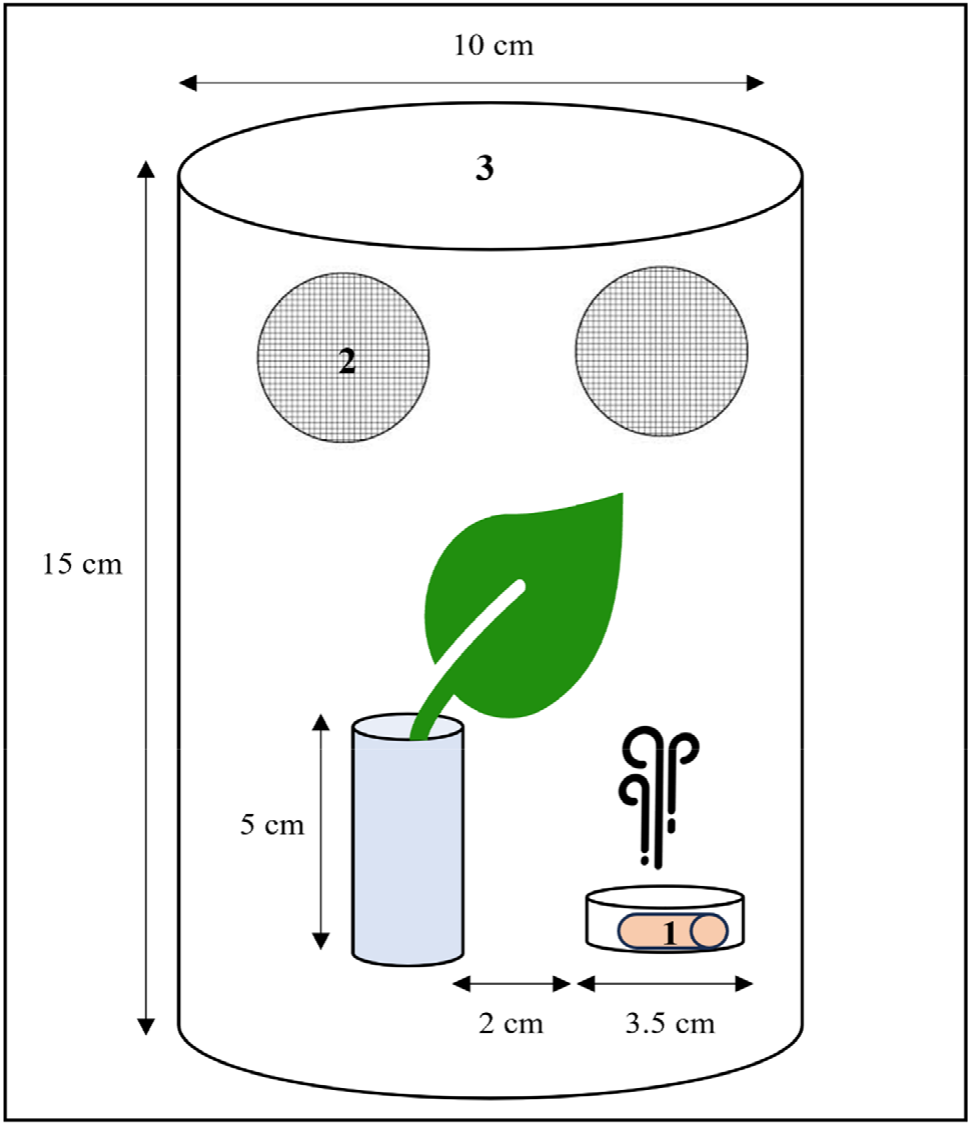
Scheme of the experimental setup. 1: cotton roll impregnated with the volatile or distilled water (control), 2: gauze‐covered openings on the cylinder, 3: transparent plastic cylinder.

In the leaf experiment without a trap, a leaf from 7‐week‐old tomato plants (*S. lycopersicum* L. cv. Brioso) was cut with its petiole, at a height of 20–50 cm above the soil. The petiole was then inserted into a glass vial (*h* × *d* = 5 cm × 2.5 cm) filled with water, to keep the leaf fresh during the experiment. A small dental cotton roll (*h* × *d* = 2 cm × 1 cm) (Nobamed Paul Danz AG, Wetter, Germany) was impregnated with 1 mL of the volatile MeSa^16^, ^34^ and placed into a Petri dish (*h* × *d* = 1 cm × 3.5 cm). Two different concentrations of the volatile were tested: a concentration 1:10 000 (*v/v*), meaning that the volatile was firstly diluted in methanol at 1:100 (*v/v*) and then diluted in distilled water at 1:100 (*v/v*), and a concentration 1:100, meaning that the volatile was firstly diluted in methanol at 1:10 (*v/v*) and then diluted in distilled water at 1:10 (*v/v*).^34^ The control consisted of 1 mL distilled water. The Petri dish was placed below the leaf, at 2 cm distance from the glass vial. A transparent plastic cylinder (*h* × *d* = 15 cm × 10 cm), with gauze‐covered openings on the sides, was used to enclose the setup (Fig. 2). About five glasshouse whiteflies were released from an Eppendorf tube (1.5 mL) inside the cylinder, 24 h prior to the introduction of the Petri dish containing the volatile, to allow for their settlement on the leaf. After this period, the whiteflies that had settled on the underside of the leaf were counted; none were found on the upper side of the leaf and whiteflies found on other surfaces were discarded. The Eppendorf tube was then manually removed, the Petri dish containing the volatile was introduced, and the plastic cylinder was replaced with an identical one which remained in place for 24 h and contained insect glue on the inside to trap dispersed whiteflies. Additionally, a black cardstock paper (size A3) was placed around each cylinder, covering its sides to minimize olfactory interference from neighboring cylinders, as well as the spread of the volatile within the cabin. Each trial lasted 24 h, and the whiteflies on the underside of the leaf were counted at 2 and 24 h from the introduction of the volatile. The whiteflies that left the underside of the leaf during a trial were considered disturbed and were found either glued on the plastic cylinder or on other surfaces within the cylinder area. To assess the amount of volatile evaporated, a random selection of cotton rolls were individually weighed pre‐ and post‐trial. After each trial, all surfaces were carefully cleaned and the insects, leaves and cotton rolls were discarded. The cylinders and Petri dishes were thoroughly washed with soapy water followed by ethanol rinsing and dried after each trial. Trials with the control were conducted in a separate climate chamber under identical conditions. A total of 40 replicates were performed, with ten replicates conducted per day every 2 days – to allow for aeration of the climate chamber – using a randomized block design.

**Figure 3:**
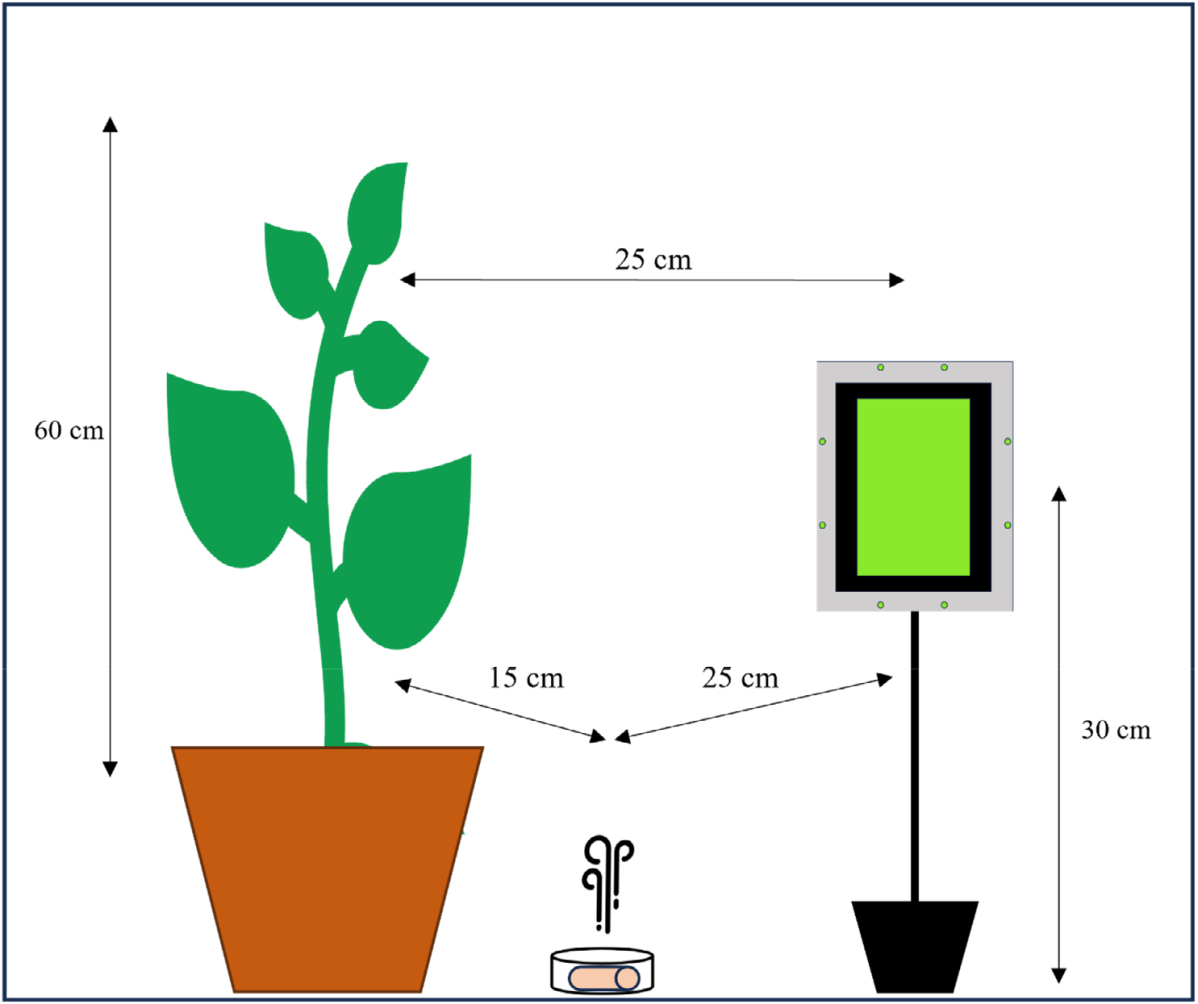
Scheme of the experimental setup including a tomato plant, a Petri dish containing the volatile, and a green light‐emitting diode (LED)‐enhanced yellow sticky trap (shown here) or a yellow sticky trap.

In a scaling‐up experiment, a 7‐week‐old tomato plant (55–60 cm) was used as whitefly host. Similarly to the previous experiment, a small dental cotton roll (*h* × *d* = 2 cm × 1 cm) was impregnated with 1 mL of the volatile and placed into a Petri dish (*h* × *d* = 1 cm × 3.5 cm). Taking into consideration that the 1:100 concentration was the most effective against whiteflies in the previous experiment, only this concentration was used in this experiment. The Petri dish was placed at 15 cm distance from the plant and 25 cm from the trap (Fig. 3). A YST or a GLT was positioned with its center at 30 cm height, facing towards the plant, with 25 cm in between. For both traps, whiteflies were caught only on the side of the trap facing towards the plant. The whole setup was enclosed in a gauze cage (90 cm × 60 cm × 60 cm). To establish the pests on the plant, 40 adult glasshouse whiteflies were released from a glass vial (*h* × *d* = 5 cm × 2.5 cm) into the cage about 24 h prior to the introduction of the Petri dish containing the volatile. After this period, the glass vial was removed and whiteflies on the plant were counted. Any whiteflies found on other surfaces were manually removed from the cage. The Petri dish containing the volatile was then introduced and remained in place for 24 h, which constituted the duration of each trial. Whiteflies on the plant and the trap were counted at 2 and 24 h from the introduction of the volatile. The whiteflies that flew off the plant were considered disturbed and were located either on the trap or on other surfaces within the cage. To assess the amount of volatile evaporated, a random selection of cotton rolls were individually weighed pre‐ and post‐trial. After counting, whiteflies, plants and cottons were discarded. The Petri dishes were thoroughly washed with soapy water followed by ethanol rinsing and dried after each trial. The same setup, without the volatile but with a trap present, was included as control and conducted in a separate climate chamber under identical conditions. A total of 15 replicates were performed, with two replicates conducted per day every 2 days – to allow for aeration of the climate chamber – using a randomized block design. Thick carton sheets (90 cm × 60 cm × 1 cm) were placed between the cages to minimize visual and volatile interference.

### 2.4 Statistical analysis

Data were statistically analyzed with R version 4.2.1 (R Core Team, 2019). The percentage of whiteflies that were disturbed, trapped out of disturbed, and trapped out of released (response variables) for different MeSa concentrations and types of traps (explanatory variables), was analyzed using generalized linear model (GLM), assuming a quasibinomial distribution (count data with overdispersion).^49^, ^50^ A deviation analysis (*F*‐test) running on the logit link was fitted to determine influences of the explanatory variables on the behavior of whiteflies settled on the host.^49^, ^51^ Subsequent Tukey‐type multiple pairwise comparisons at *α* = 0.05 using the R‐package ‘emmeans’^52^ were conducted to clarify which treatment differed from another (mean value differences) in each experiment. All figures showing results are boxplots and were made using R (version 4.2.1) and the ggplot2 package.^53^

## 3 RESULTS

### 3.1 Effect of MeSa on whitefly dispersal from a leaf (without a trap)

**Figure 4:**
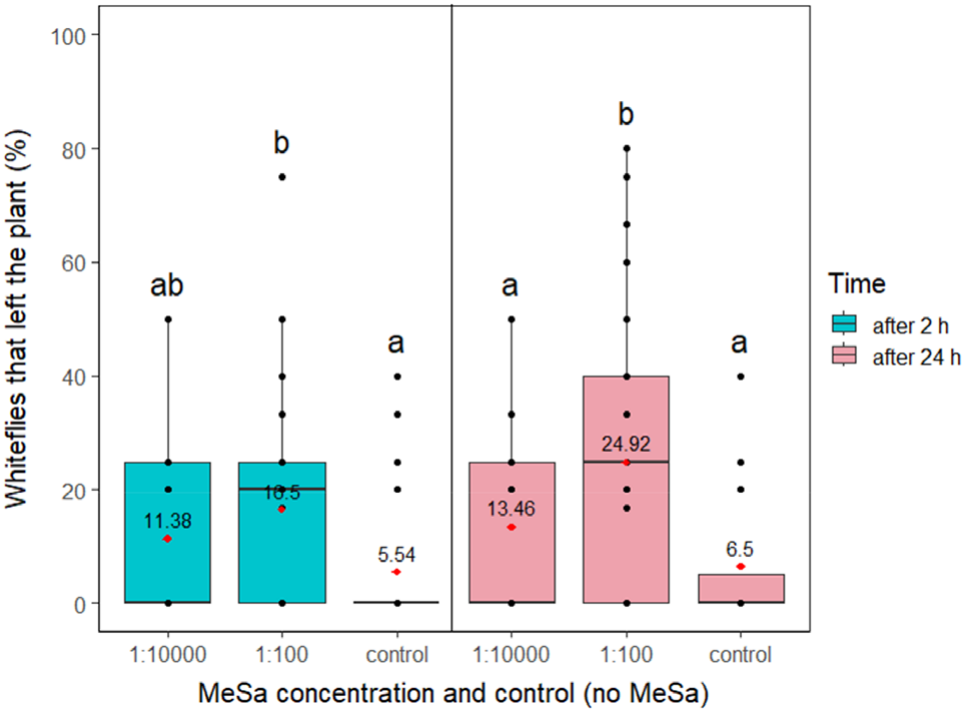
Disturbed whiteflies that left the underside of the leaf in no‐choice experiments under two different methyl salicylate (MeSa) concentrations, after 2 and 24 h of exposure to the volatile, in controlled conditions. Means are indicated with red dots and labeled with numbers. Significant differences between the effect of different treatments are represented by different letters (GLM, pairwise mean comparisons, *α* = 0.05).

From the initial release of approximately five whiteflies, an average of 4.2 were settled on the underside of leaves before introduction of MeSa. The experiment demonstrated that MeSa had a significant effect on the disturbance of whiteflies after 2 h (*F*_2,119_ = 5.56, *P* = 0.005) and 24 h (*F*_2,119_ = 9.91, *P* < 0.001). In the first 2 h, the volatile in higher concentration (1:100) significantly differed from the control, disturbing nearly three times as many whiteflies (*P* = 0.005). The lower concentration of the volatile (1:10 000) disturbed two times more whiteflies than the control, but this difference was not significant (*P* = 0.12). After 24 h, whitefly disturbance under the higher volatile concentration increased by 8.42%, reaching nearly 25% of disturbed whiteflies in total. This was 3.83 and 1.85 times higher than the control (*P* < 0.001) and the lower concentration (*P* = 0.01), respectively. The lower volatile concentration increased the disturbed whiteflies by two‐fold compared to the control, but the difference was not significant (*P* = 0.26) (Fig. 4). The amount of volatile that had evaporated from a single cotton roll was 0.05 mL after 2 h and 0.83 mL after 24 h.

### 3.2 Effect of MeSa on whitefly dispersal from a plant (with a trap)

**Figure 5:**
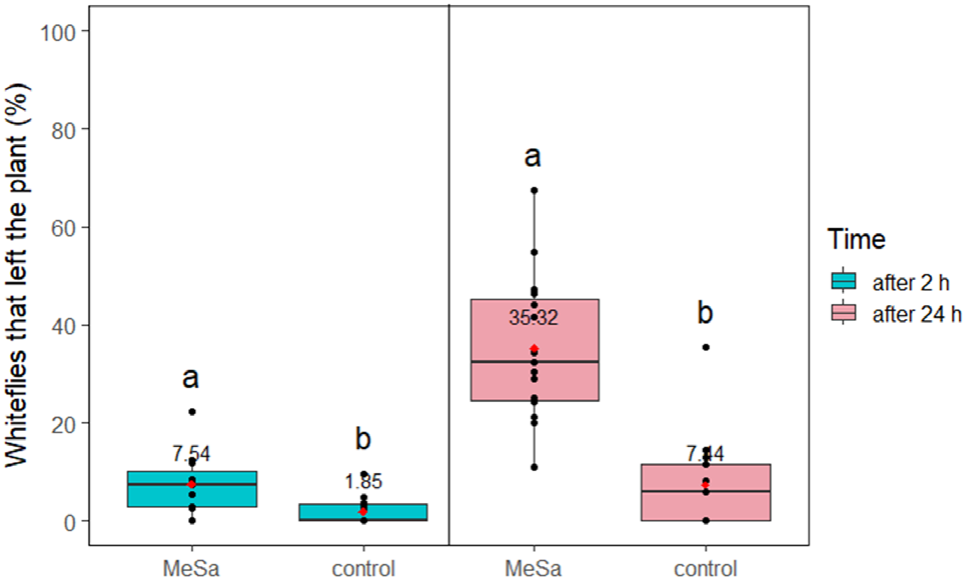
Disturbed whiteflies that left the tomato plant after 2 and 24 h of exposure to 1:100 methyl salicylate (MeSa) and the control (no MeSa), with a yellow sticky trap present, in controlled conditions. Means are indicated with red dots and labeled with numbers. Significant differences between the effect of different treatments are represented by different letters (GLM, pairwise mean comparisons, *α* = 0.05).

**Figure 6:**
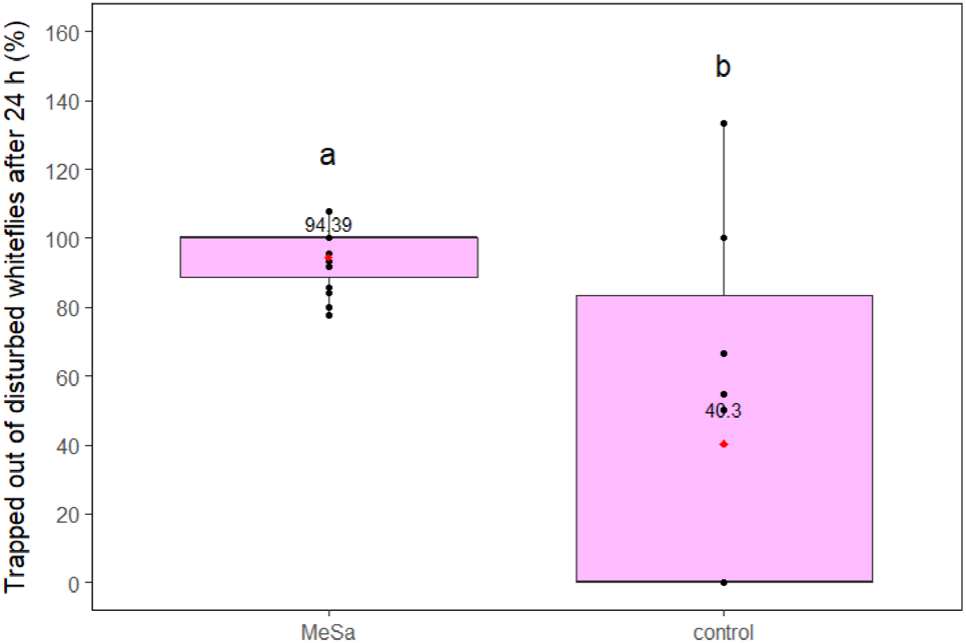
Whiteflies caught on a yellow sticky trap after they left the tomato plant, during 24 h of exposure to 1:100 methyl salicylate (MeSa) and the control (no MeSa), under controlled conditions. Means are indicated with red dots and labeled with numbers. Significant differences between the effect of different treatments are represented by different letters (GLM, pairwise mean comparisons, *α* = 0.05).

The results showed that the volatile had an effect on whitefly disturbance regardless of the trap type. Specifically, in presence of a YST, MeSa had a highly significant effect on the disturbance of whiteflies after 2 h (*F*_1,29_ = 14.67, *P* < 0.001) and also after 24 h (*F*_1,29_ = 34.61, *P* < 0.001). The volatile disturbed 4 and 4.7 times more whiteflies compared to the control after 2 and 24 h, respectively (both *P* < 0.001), disturbing 35.32% of whiteflies in total (Fig. 5). The percentage of whiteflies trapped on the YST after leaving the plant was evaluated only after 24 h of exposure to the volatile or the control. MeSa significantly affected this percentage (*F*_1,29_ = 6.29, *P* = 0.02), with 94.39% of the whiteflies being trapped in the presence of the volatile, which was 2.34 times higher than the control (*P* = 0.008) (Fig. 6).

**Figure 7:**
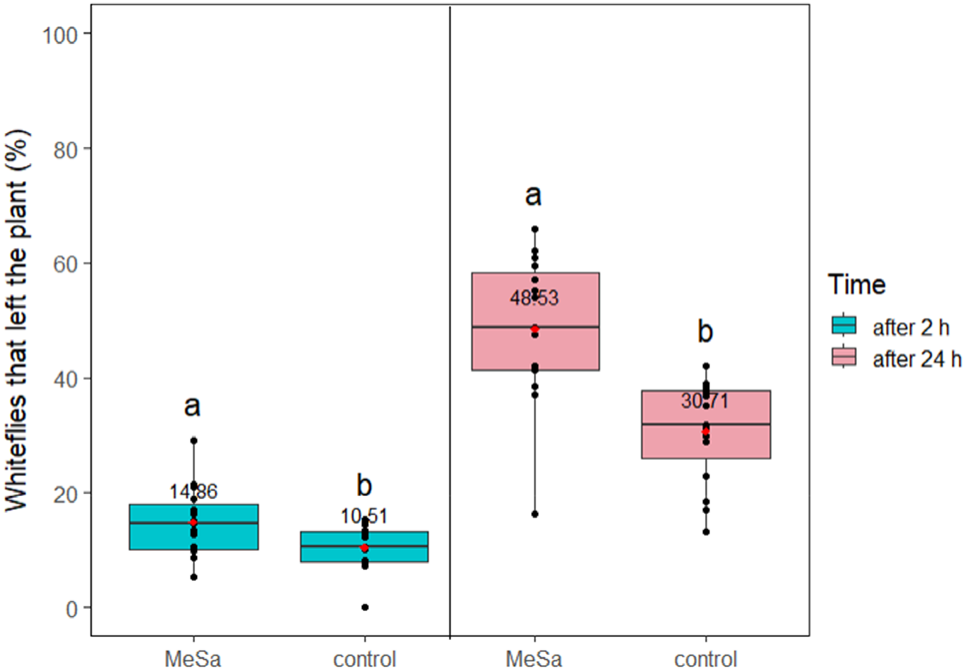
Disturbed whiteflies that left the tomato plant after 2 and 24 h of exposure to 1:100 methyl salicylate (MeSa) and the control (no MeSa), with a green light‐emitting diode (LED)‐enhanced yellow sticky trap present, in controlled conditions. Means are indicated with red dots and labeled with numbers. Significant differences between the effect of different treatments are represented by different letters (GLM, pairwise mean comparisons, *α* = 0.05).

**Figure 8:**
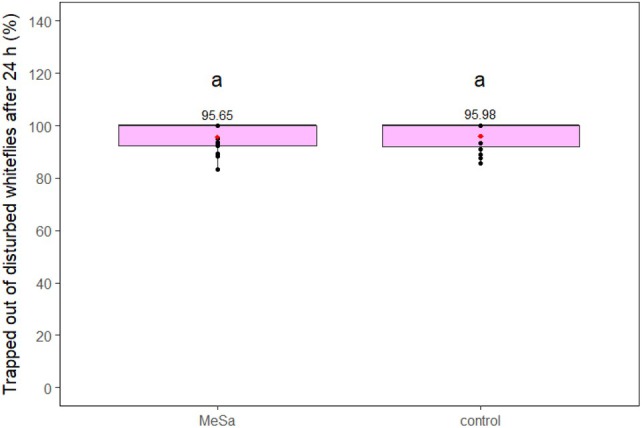
Whiteflies caught on a green light‐emitting diode (LED)‐enhanced yellow sticky trap after they left the tomato plant, during 24 h of exposure to 1:100 methyl salicylate (MeSa) and the control (no MeSa), under controlled conditions. Means are indicated with red dots and labeled with numbers. Significant differences between the effect of different treatments are represented by different letters (GLM, pairwise mean comparisons, *α* = 0.05).

In presence of a GLT, MeSa had a significant effect on the whiteflies leaving the plant after 2 h (*F*_1,29_ = 5.81, *P* = 0.02) and 24 h (*F*_1,29_ = 19.55, *P* < 0.001). The volatile disturbed 1.4 and 1.6 times more whiteflies compared to the control after 2 and 24 h, respectively (both *P* < 0.05), resulting in a total whitefly disturbance of 48.53% (Fig. 7). The percentage of whiteflies trapped on the GLT after leaving the plant was evaluated only after 24 h of exposure to the volatile or the control. In contrast to the results observed with the YST, MeSa did not significantly influence this percentage (*F*_1,29_ = 0.03, *P* = 0.85), as more than 95% of the disturbed whiteflies were trapped both in presence of the volatile and the control (*P* = 0.85) (Fig. 8).

**Figure 9:**
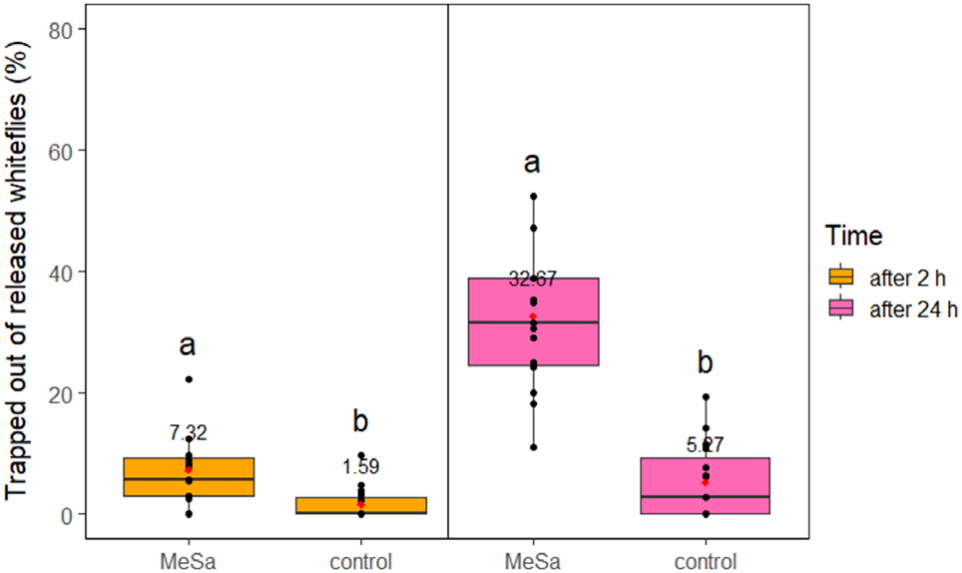
Whiteflies caught on yellow sticky trap from the initially released population after 2 and 24 h of exposure to 1:100 methyl salicylate (MeSa) and the control (no MeSa), in controlled conditions. Means are indicated with red dots and labeled with numbers. Significant differences between the effect of different treatments are represented by different letters (GLM, pairwise mean comparisons, *α* = 0.05).

**Figure 10:**
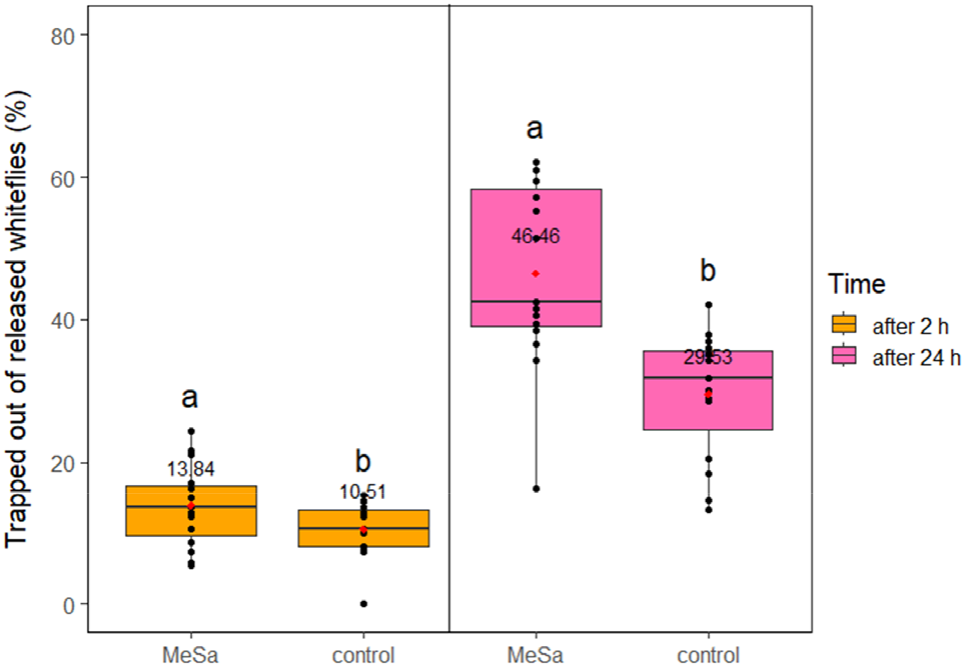
Whiteflies caught on green light‐emitting diode (LED)‐enhanced yellow sticky trap from the initially released population after 2 and 24 h of exposure to 1:100 methyl salicylate (MeSa) and the control (no MeSa), in controlled conditions. Means are indicated with red dots and labeled with numbers. Significant differences between the effect of different treatments are represented by different letters (GLM, pairwise mean comparisons, *α* = 0.05).

**Figure 11:**
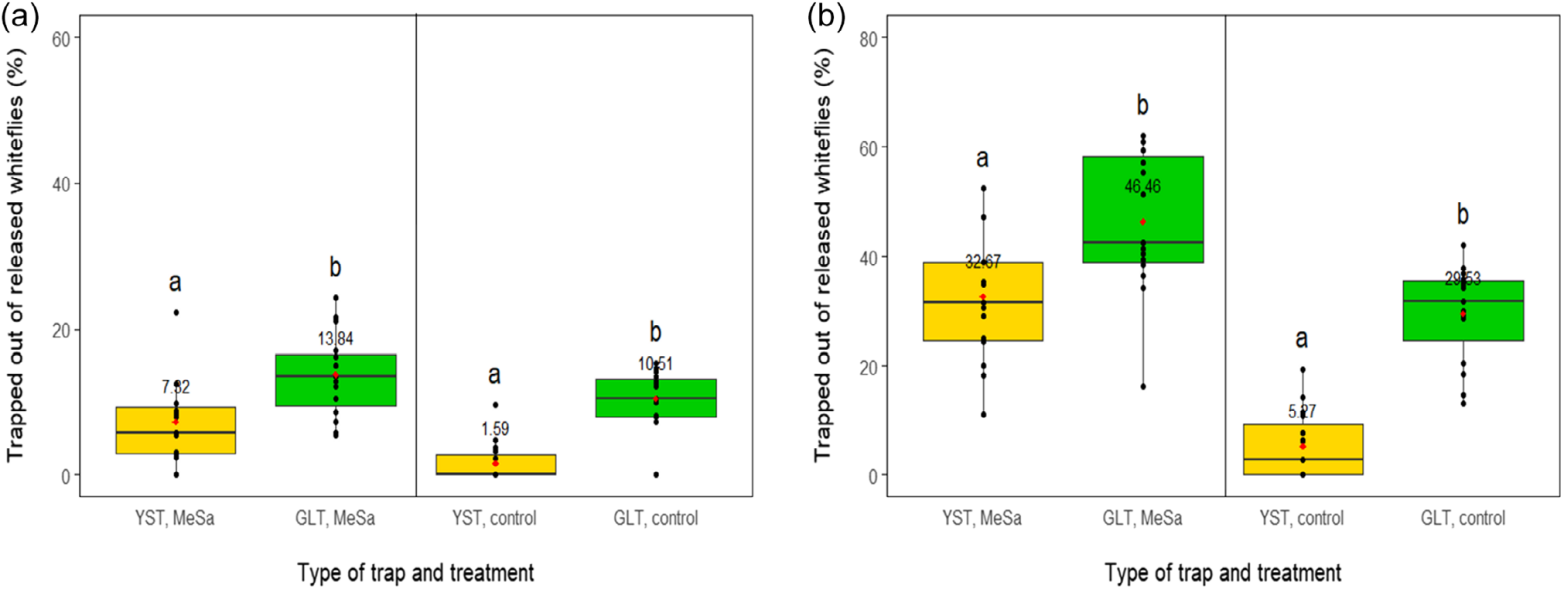
Whiteflies caught on a yellow sticky trap (YST) or a green light‐emitting diode (LED)‐enhanced yellow sticky trap (GLT) from the initially released population after 2 h (a) and 24 h (b) of exposure to 1:100 methyl salicylate (MeSa) and the control (no MeSa), in controlled conditions. Means are indicated with red dots and labeled with numbers. Significant differences between the effect of different traps are represented by different letters (GLM, pairwise mean comparisons, *α* = 0.05).

MeSa had an effect on the trapped out of released whiteflies as both traps captured notably more whiteflies in presence of the volatile compared to the control after 2 h (YST: *F*_1,29_ = 14.65, *P* < 0.001, GLT: *F*_1,29_ = 3.91, *P* = 0.05) and 24 h (YST: *F*_1,29_ = 50.28, *P* < 0.001, GLT: *F*_1,29_ = 18.62, *P* < 0.001). Specifically, after 24 h, the YST captured 32.67% of the released whiteflies, which was six times more than the control (*P* < 0.001) (Fig. 9) and the GLT captured 46.46% of the released whiteflies, which was 1.6 times more than the control (*P* < 0.001) (Fig. 10). Moreover, the type of the trap had an effect on the capture of the released whiteflies in presence of the volatile and the control, as the GLT captured significantly more whiteflies than the YST after 2 h (MeSa: *F*_1,29_ = 9.66, *P* = 0.004, control: *F*_1,29_ = 40.77, *P* < 0.001) and 24 h (MeSa: *F*_1,29_ = 7.51, *P* = 0.01, control: *F*_1,29_ = 53.63, *P* < 0.001) (Fig. 11). The amount of volatile that evaporated from a single cotton roll was 0.13 mL after 2 h and 1 mL after 24 h.

## 4 DISCUSSION

The findings of this study reveal that MeSa can effectively disturb *T. vaporariorum* settled on a host plant, thereby increasing the likelihood that the dispersed whiteflies will land on an attractive trap. Furthermore, our results confirm that the GLT exhibits higher attractivity than the YST, and provide additional evidence that the presence of MeSa enhances the efficacy of both traps.

When we tested the behavior of whiteflies settled on a single leaf, MeSa significantly disrupted whitefly behavior compared to the control, already within the first 2 h, but only at the higher volatile concentration. In contrast, the lower concentration disturbed 1.45 times more whiteflies compared to the control, but the difference was not statistically significant. This indicates that the disturbance effect is dependent on the MeSa concentration, aligning with the established principle that volatile repellency exhibits a positive correlation with increasing concentration.^54^ Moreover, our findings are consistent with those of Ali *et al*.,^55^ who showed reduced settlement and survival of peach potato aphids (*Myzus persicae*) on plants treated with higher MeSa concentrations compared to those with lower concentrations, revealing a concentration‐dependent effect in aphid performance on *Brassica* plants. Our experiment validates this effect both after 2 and 24 h of exposure to the two different MeSa concentrations. Previous research has demonstrated that the presence of MeSa reduced the performance of pests on plants by stimulating the production of plant volatile compounds that activate defense mechanisms against pests and also by attracting natural enemies.^31^, ^55^, ^56^, ^57^ Shulaev *et al*.^22^ indicated that MeSa is involved in SAR signaling, showing that MeSa moves from infected sites of the plant to uninfected ones, where it gets converted back to SA, initiating defense responses throughout the plant. The results of our experiment extend these findings by demonstrating that MeSa, even as an isolated volatile, can directly induce whitefly disturbance, since the use of a single leaf precludes the activation of plant defense responses through SAR.^58^, ^59^ This suggests that MeSa may have a dual role in whitefly control, both indirectly through plant‐mediated mechanisms and directly through its disturbing effect on whiteflies.

When we tested the behavior of whiteflies settled on a plant, the results showed that MeSa disturbed significantly more whiteflies compared to the control, regardless of using a YST or a GLT. The fact that this effect occurred within the first 2 h further underscores that MeSa alone can induce whitefly dispersal, since activation of plant defense mechanisms is reported to take place after 24–48 h of exposure to MeSa.^34^, ^55^, ^56^, ^60^ Moreover, glasshouse whitefly population development decreased on plants that were treated with MeSa for 5 days and then kept for an additional 10 days to allow activation of plant defenses before the whiteflies were introduced.^35^ This could also explain why whitefly disturbance increased 4.7 and 3.3 times between 2 and 24 h in presence of a YST and a GLT, respectively. During this period, production of VOCs that activate plant resistance and therefore repel whiteflies was initiated by the plant, leading to the enhanced expression of defensive genes, which subsequently deterred the whiteflies.^34^

While previous studies showed that MeSa can repel whiteflies and other pests when their introduction occurs after the plants have already been MeSa‐treated, and therefore used MeSa as a preventive control method, our results demonstrate that MeSa activated significantly more whiteflies already settled on tomato plants compared to non‐MeSa‐treated plants. This reveals the possibility to use this volatile as a curative control measure, especially in the early stages of whitefly infestation (as in our experiment), indicating that MeSa can induce dispersal even in scenarios of low pest density and high plant quality. Given that whiteflies were already settled and feeding on leaves, it can be proposed that the interference of MeSa overrides the feeding/oviposition signal, even when the whitefly's piercing mouthparts are already inserted into the leaf tissue. A 2‐h exposure to MeSa resulted in a significantly higher number of dispersed whiteflies compared to the control, indicating the pest's ability to perceive the volatile soon after exposure to it. Beyond this 2 h threshold, MeSa clearly signals either poor host plant quality or potential threat, prompting the whiteflies to leave their valuable resource in search of an alternative, regardless of feeding or oviposition. This underscores the significance of olfactory cues in triggering behavioral responses in whiteflies over short distances, a phenomenon that is similarly observed in other insects.^61^, ^62^, ^63^

In presence of MeSa, both the YST and GLT captured a significantly higher number of released whiteflies compared to the control. This indicates that presence of this volatile not only induced whitefly flight behavior but also increased their probability to get caught on a trap – an attractive visual stimulus within their environment. Significantly higher number of released whiteflies were attracted to the GLT than the YST in the first 2 h, supporting previous findings of whiteflies showing higher preference for GLT than YST after 2 h of exposure to them.^47^, ^64^ Moreover, the significantly higher catching rate of the GLT compared to the YST in the control highlights the GLT's ability to also initiate whitefly movement, making it an appealing alternative for whiteflies already settled on the canopy. Upon taking flight, whiteflies are more inclined to land on a surface that emits light at 520 nm, closely mimicking the peak reflectance of green leaves, as opposed to a YST that reflects a wider spectrum of light.^45^, ^47^ This preference, observed in the small whitefly population in our experiment, along with the GLT's ability to maintain consistent light intensity regardless of sunlight conditions,^65^ suggests that YSTs enhanced with green LEDs could be effective for monitoring and/or mass trapping in the glasshouse when whitefly populations are still low. However, the GLT demonstrated greater attractiveness compared to the YST even under conditions of high whitefly populations in the glasshouse.^47^ Additionally, the observation that over 95% of the whiteflies that took flight from the plant did not return, but instead landed on the GLT, irrespective of the treatment, confirms the trap's property to attract whiteflies once they have left the plant, as seen also in previous studies under glasshouse conditions.^47^ This also demonstrates its strong attraction effect, even with the plant canopy present.

When whitefly behavior was tested on a plant, it was visually observed that more whiteflies took off from the lower parts of the plant compared to the higher ones (data not shown). This indicates that placing the volatile at both the lower and upper levels of the plant canopy could potentially increase disturbance rates. Furthermore, targeting the upper plant parts with the volatile is likely to enhance whitefly disturbance, given that whiteflies frequently migrate to younger plant tissues for oviposition,^66^, ^67^ where they would be exposed to the volatile post‐emergence. Furthermore, repeated applications of MeSA could gradually reduce whitefly population growth overtime. By disturbing adults and interrupting oviposition, it could limit the development of new generations, contributing to long‐term pest control. Moreover, combining MeSa with visual cues – such as blue LEDs – may enhance efficacy against whiteflies, as suggested by our preliminary trials (unpublished data). These insights open opportunities to tailor pest deterrents by adjusting cue combinations, intensity, and duration. For practical implementation, efficient and targeted MeSa release systems are needed; smart dispensers integrated with sensors could optimize application by responding to pest presence or trap position in real time. However, validation under glasshouse and field conditions is essential to assess MeSa's performance under variable environmental factors, including possible challenges such as MeSa evaporating too quickly to remain effective. With further development, MeSa‐based technologies could reduce chemical inputs and contribute to more sustainable whitefly management.

## 5 CONCLUSION

Our research demonstrated that MeSa effectively deters whiteflies from their host and increases the capture rates of both a YST and a GLT, suggesting a novel push–pull approach in integrated pest management (IPM). Due to the absence of other olfactory influences in the climate chamber, the results provide valuable insights into the sensitivity of whiteflies to this volatile compound. The ability of MeSa to disturb nearly 50% of settled whiteflies on a plant underscores its potential as an effective tool for whitefly biocontrol, especially during the initial stages of infestation, when the need for dispersal movements is minimal. The enhanced effectiveness of the GLT over the conventional YST in capturing whiteflies highlights its ability for effective monitoring and potential for mass trapping when combined with MeSa as a deterrent factor. Considering that MeSa contributes to inducing SAR, indirect defense responses in tomato plants when attacked by herbivores, and attracting natural enemies,^25^, ^33^, ^36^ its application within a crop is expected to be beneficial rather than detrimental to the plants. Future research should aim to determine whether this innovative technology‐based push–pull approach is effective under glasshouse growing conditions and whether pest population density can be kept below economic thresholds for longer periods of time.

## FUNDING INFORMATION

This work was supported by funds of the Federal Ministry of Food and Agriculture (BMEL) based on a decision of the Parliament of the Federal Republic of Germany *via* the Federal Office for Agriculture and Food (BLE) under the innovation support program ptble (Projektträger Bundesanstalt für Landwirtschaft und Ernährung).

## CONFLICT OF INTEREST

The authors declare that they have no known competing financial interests or personal relationships that could have appeared to influence the work reported in this paper.

## AUTHOR CONTRIBUTIONS

MA: conceived and designed the experiments, conducted the experiments, analyzed data, wrote draft manuscript, did review and editing. R.M: provided resources, supervision, project administration, acquired funding, designed the experiments, did data supervision, review and editing. All of the authors read and approved the manuscript.

## Data Availability

https://doi.org/10.25835/bbt5vgdo.^68^
